# A new method to manipulate broiler chicken growth and metabolism: Response to mixed LED light system

**DOI:** 10.1038/srep25972

**Published:** 2016-05-12

**Authors:** Yefeng Yang, Yonghua Yu, Jinming Pan, Yibin Ying, Hong Zhou

**Affiliations:** 1 College of Biosystems Engineering and Food Science, Zhejiang University, Hangzhou 310058, China; 2 Department of Instrument Science and Engineering, Zhejiang University, Hangzhou 310058, China

## Abstract

Present study introduced a new method to manipulate broiler chicken growth and metabolism by mixing the growth-advantage LED. We found that the green/blue LED mixed light system (G-B and G × B) have the similar stimulatory effect on chick body weight with single green light and single blue light (G and B), compared with normal artificial light (*P* = 0.028). Moreover, the percentage of carcass was significantly greater in the mixed light (G × B) when compared with the single light (*P* = 0.003). Synchronized with body weight, the mixed light (G-B and G × B) had a significant improved influence on the feed conversion of birds compared with normal light (*P* = 0.002). A significant improvement in feed conversion were found in mixed light (G × B) compared with single LED light (*P* = 0.037). G group resulted in a greater high-density lipoprotein cholesterol level than B group (*P* = 0.002), whereas B group resulted in a greater low-density lipoprotein cholesterol level than G group (*P* = 0.017). The mixed light significantly increased the birds’ glucose level in comparison with the single light (*P* = 0.003). This study might establish an effective strategy for maximizing growth of chickens by mixed LED technology.

With impacts of climate change, issues such as more frequent and serious droughts, floods, and storms as well as diseases are becoming more serious threats to agriculture. These threats along with shortage of food supply make people turn to indoor and urban farming (such as vertical farming) for help. With proper lighting, indoor agriculture eliminates weather-related crop failures to provide year-round agriculture production, which assist in supplying food in cities with surging populations and in areas of severe environmental conditions. Therefore, light supplementation serve as a powerful tool in agriculture production^1^,^2^,^3^.

Previous studies indicate that light play a vital role in affecting function and behavior of chickens^4^,^5^,^6^,^7^. Further it has been reported that light manipulation has been an effective measure to improve poultry production^8^,^9^,^10^. On the one hand, poultry have highly specialized visual systems and the majority of their behavior is mediated by vision^11^. The poultry retina possesses one of the most sophisticated cone photoreceptor systems among vertebrates. Poultry have five types of cones, including four single cones that support tetrachromatic color vision and one double cone, which is thought to mediate achromatic motion perception. Tetrachromatic color vision is mediated by four types of single cones that are maximally responsive to violet, blue, green and red light^12^. Second, light is in control of many physiological and behavioral processes of poultry. Circadian regulation of energy homeostasis is controlled by an endogenous biological clock, located in the suprachiasmatic nuclei (SCN) of the hypothalamus, that is synchronized by photic information that travels directly from light-sensitive ganglion cells in the retina to the SCN, thereby entraining individuals’ physiology and behavior to the ambient light^13^. Importantly, light is a powerful exogenous factor entraining signal for circadian clock, although other factors such as food consumption influence clock signaling^13^. Therefore, light plays a vital role in affecting the growth and the development of poultry.

The only light source for chickens in an environmentally controlled chicken house is artificial light. Thus, the light wavelength (quality), the light intensity (quantity), and the light period (regime) have become major factors in modern poultry management. Among all light sources, light-emitted dioxide (LED) is a unique type of semiconductor diode. Compare to incandescent light’s 1000-h and fluorescent light’s 8000-h life span, LEDs have a very significantly longer life of 100,000 h. In addition to their long life, LEDs have many advantages over conventional light source. These advantages include small size, specific wavelength, low thermal output, adjustable light intensity and quality, as well as high photoelectric conversion efficiency. Such advantages make LEDs perfect for supporting chick growth in controlled environment. Studies in our laboratory indicated that light period^9^ and light intensity^14^ emitted by LEDs showed a significant influence on the growth and behavior of the chicken broilers. Further we reported that monochromatic LED light also can affect growth of the chicken broilers^15^. Especially, short wavelength light (monochromatic green LED light and blue LED light) have been reported to stimulate growth of broilers compare with normal white light^15^.

In present study, we introduced a new method to manipulate chick growth and metabolism by mixing the growth-advantage LED (monochromatic green LED light and blue LED light). First, the green LED chip and blue LED chip have been combined to fabricate the mixed LED arrays; second, the relationships between the mixed light spectra and growth performance as well as metabolic response of chicks have been investigated.

## Results

### Growth performance

**Figure 1:**
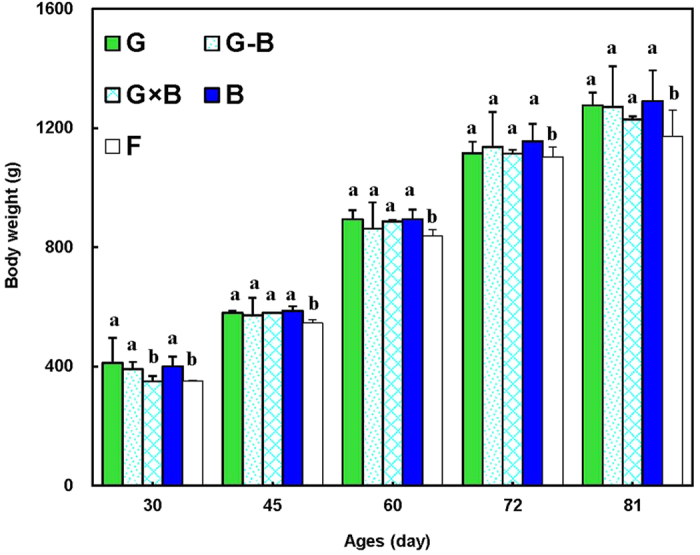
Body weight (g) of broilers reared under different lighting system. Each group of treated birds was reared under either cell (replicate) equipped with two green LED lights (G group), cell (replicate) equipped with a green LED light and a blue LED light (G-B group), cell (replicate) equipped with two green-blue-dual-wavelength LED lights (G × B group), cell (replicate) equipped with two blue LED lights (B group), or cell (replicate) equipped with two normal artificial lights (F group as control group; fluorescent lamps) from 1 day old until termination of experiment at 81 day of age. Body weight was measured at 30, 45, 60, 72 and 81 day of age. Data are expressed as mean value ± SEM. Bars marked with different letters are significantly different from each other (*P* < 0.05).

**Figure 2:**
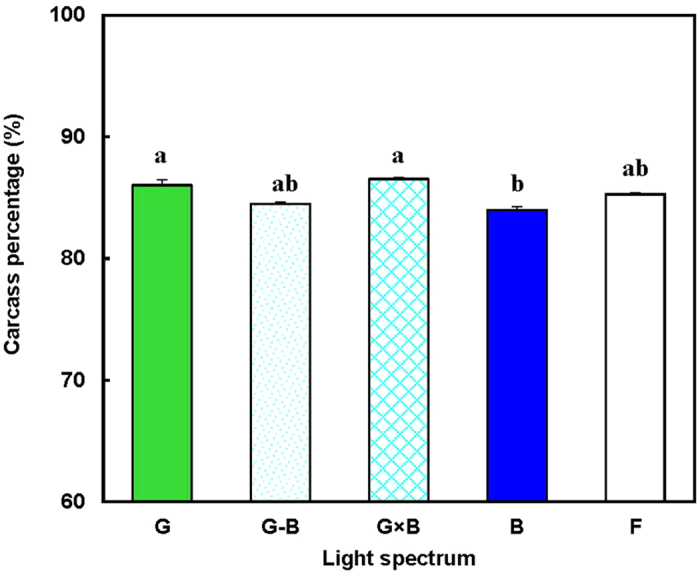
Carcass percentage of 81-day-old broilers reared under different lighting system. Each group of treated birds was reared under either cell (replicate) equipped with two green LED lights (G group), cell (replicate) equipped with a green LED light and a blue LED light (G-B group), cell (replicate) equipped with two green-blue-dual-wavelength LED lights (G × B group), cell (replicate) equipped with two blue LED lights (B group), or cell (replicate) equipped with two normal artificial lights (F group as control group; fluorescent lamps) from 1 day old until termination of experiment at 81 day of age. At the end of the trial (81 day of age), after being fasted for 12 h, birds from each replicate were killed by exsanguination, plucked, and eviscerated to measure weights of carcass. Carcass percentage = carcass weight/body weight. Data are expressed as mean value ± SEM. Bars marked with different letters are significantly different from each other (*P* < 0.05).

The effects of the green/blue mixed LED light system (G-B and G × B groups) on the body weight of the broilers are shown in Fig. 1. The green/blue LED mixed light system (G-B and G × B) have the same stimulatory effect on broiler body weight with single green light and single blue light (G and B groups), compared with normal artificial light (F group, *P* = 0.028). Additionally, no significant differences were found the body weight of birds between the mixture of pure green light and pure blue light (G-B and G × B groups) and single pure green or blue light except on 30 days of age. In the early days of the study (30 days), a significant decrease in the body weight of the birds reared in the G × B group was observed compared with the G group and the B group (*P* = 0.003). In the older broilers (after 45 days), no significant difference in the body weight was found between a single pure light and the mixed lights (*P* = 0.153). Moreover, the percentage of carcass was significantly higher in the G group and the G × B group when compared with the B group (*P* = 0.003) (Fig. 2).

**Figure 3:**
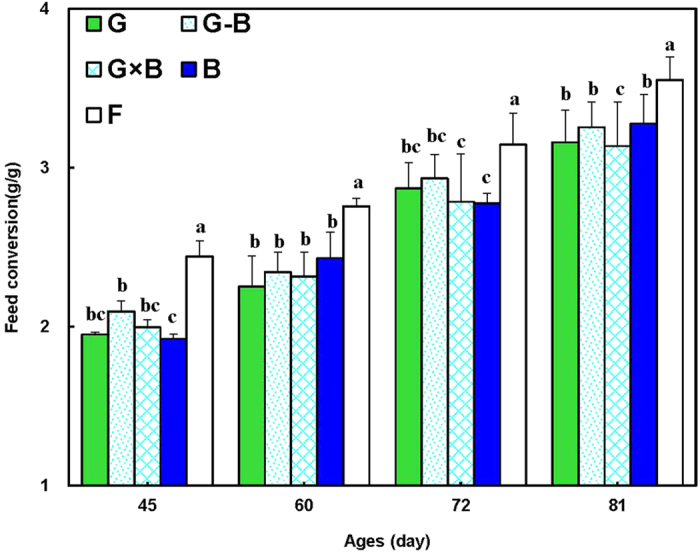
Feed conversion (g/g) of broilers reared under different lighting system. Each group of treated birds was reared under either cell (replicate) equipped with two green LED lights (G group), cell (replicate) equipped with a green LED light and a blue LED light (G-B group), cell (replicate) equipped with two green-blue-dual-wavelength LED lights (G × B group), cell (replicate) equipped with two blue LED lights (B group), or cell (replicate) equipped with two normal artificial lights (F group as control group; fluorescent lamps) from 1 day old until termination of experiment at 81 day of age. Cumulative feed consumption and body weight was measured at 35, 49, 63 and 80 day of age to calculate the feed conversion. Feed conversion = (finial period weight – initial period weight)/period feed consumption. Data are expressed as mean value ± SEM. Bars marked with different letters are significantly different from each other (*P* < 0.05).

With the growth and development of the broilers, the consumption of feed increased gradually. As shown in Fig. 3, the single LED lights (G and B groups) and green/blue mixed LED lights (G-B and G × B groups) had a significant improved influence on the feed conversion of birds compared with normal artificial light (F group) (*P* = 0.002). Moreover, at the end of the study (81 days), a significant improvement in feed conversion were found in G × B group compared with all others groups (*P* = 0.037).

**Figure 4:**
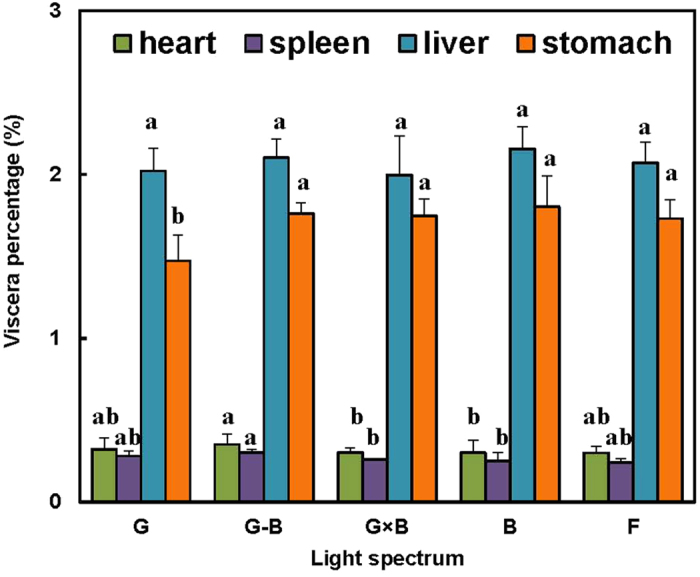
Viscera weight percentage of 81-day-old broilers reared under different lighting system. Each group of treated birds was reared under either cell (replicate) equipped with two green LED lights (G group), cell (replicate) equipped with a green LED light and a blue LED light (G-B group), cell (replicate) equipped with two green-blue-dual-wavelength LED lights (G × B group), cell (replicate) equipped with two blue LED lights (B group), or cell (replicate) equipped with two normal artificial lights (F group as control group; fluorescent lamps) from 1 day old until termination of experiment at 81 day of age. At the end of the trial (81 day of age), after being fasted for 12 h, birds from each replicate were killed by exsanguination, plucked, and eviscerated to measure weights of heart and liver. Viscera percentage = viscera weight/body weight. Data are expressed as mean value ± SEM. Bars marked with different letters are significantly different from each other (*P* < 0.05).

No significant differences were found for the heart, the spleen, or the liver weight between the birds treated with a single treatment (G and B groups) or mixed lights treatment (G-B and G × B groups) and the birds with normal artificial lights treatment (*P* = 0.256) (Fig. 4). However, the birds treated with the G-B group had the greater heart and spleen weights than B group (*P* = 0.026 and 0.031). The birds treated with the G-B group and G × B group had the greater stomach weight than G group (*P* = 0.011 and 0.0019). Additionally, no significant differences either between between G-B group and G × B group either in the heart, the spleen, the liver weight, or the stomach weights (*P* = 0.167).

**Figure 5:**
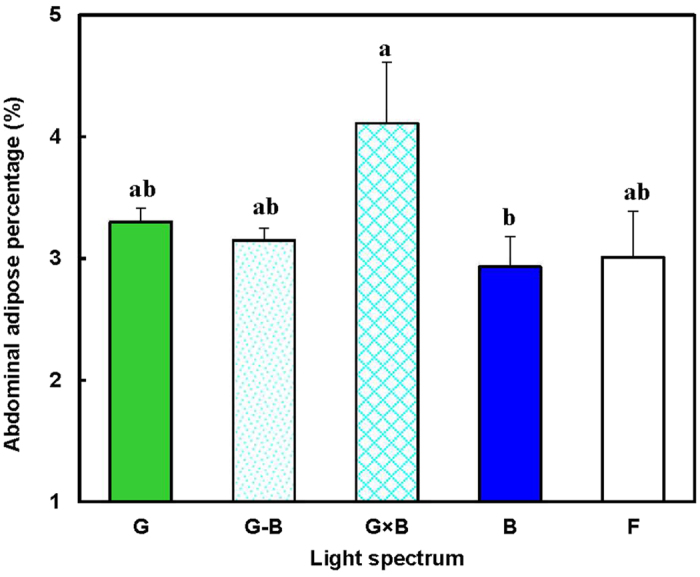
Abdominal adipose weight percentage of 81-day-old broilers reared under different lighting system. Each group of treated birds was reared under either cell (replicate) equipped with two green LED lights (G group), cell (replicate) equipped with a green LED light and a blue LED light (G-B group), cell (replicate) equipped with two green-blue-dual-wavelength LED lights (G × B group), cell (replicate) equipped with two blue LED lights (B group), or cell (replicate) equipped with two normal artificial lights (F group as control group; fluorescent lamps) from 1 day old until termination of experiment at 81 day of age. At the end of the trial (81 day of age), after being fasted for 12 h, birds from each replicate were killed by exsanguination, plucked, and eviscerated to measure weights of abdominal adipose. Adipose weight percentage = adipose weight/body weight. Data are expressed as mean value ± SEM. Bars marked with different letters are significantly different from each other (*P* < 0.05).

Furthermore, a significant increase in the abdominal adipose tissue weight was found in birds treated with the G × B group compared with the birds treated with B group (*P* = 0.021) (Fig. 5). Whereas, no significant difference in this parameter was observed among the G, B, G-B and F groups (*P* = 0.605).

### Metabolic indicators

**Figure 6:**
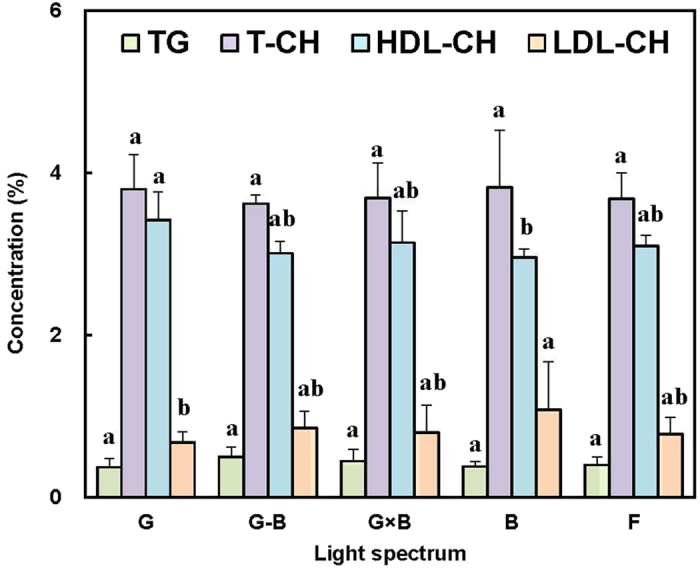
Plasma biochemistry parameters (mmol/L) of 81-day-old broilers reared under different lighting system. Each group of treated birds was reared under either cell (replicate) equipped with two green LED lights (G group), cell (replicate) equipped with a green LED light and a blue LED light (G-B group), cell (replicate) equipped with two green-blue-dual-wavelength LED lights (G × B group), cell (replicate) equipped with two blue LED lights (B group), or cell (replicate) equipped with two normal artificial lights (F group as control group; fluorescent lamps) from 1 day old until termination of experiment at 81 day of age. At the end of the trial (81 day of age), after being fasted for 12 h, birds from each replicate were killed cervical dislocation to collect blood samples. Total cholesterol (TC), total triglyceride (TG), high density lipoprotein cholesterol (HDL-CH) and low density lipoprotein cholesterol (LDL-CH) were determined. Data are expressed as mean value ± SEM. Bars marked with different letters are significantly different from each other (*P* < 0.05).

**Figure 7:**
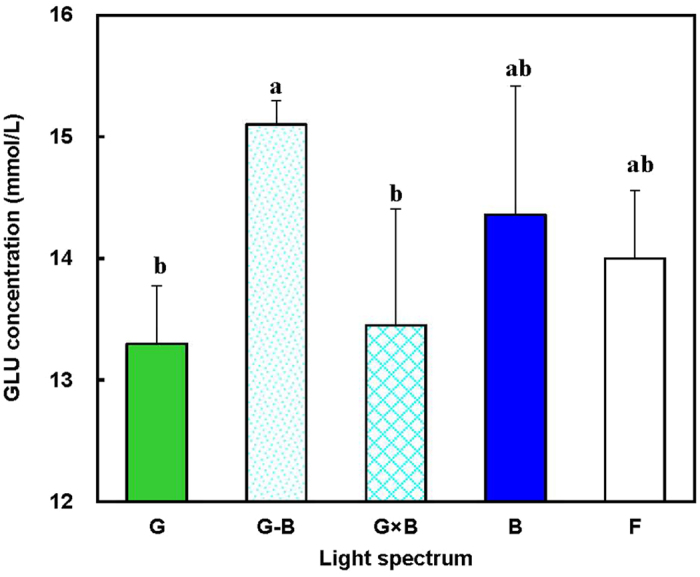
Glucose (GLU) level (mmol/L) of 81-day-old broilers reared under different lighting system. Each group of treated birds was reared under either cell (replicate) equipped with two green LED lights (G group), cell (replicate) equipped with a green LED light and a blue LED light (G-B group), cell (replicate) equipped with two green-blue-dual-wavelength LED lights (G × B group), cell (replicate) equipped with two blue LED lights (B group), or cell (replicate) equipped with two normal artificial lights (F group as control group; fluorescent lamps) from 1 day old until termination of experiment at 81 day of age. At the end of the trial (81 day of age), after being fasted for 12 h, birds from each replicate were killed cervical dislocation to collect blood samples. Data are expressed as mean value ± SEM. Bars marked with different letters are significantly different from each other (*P* < 0.05).

As shown in Fig. 6, there were no significant differences found among the treatments for the blood biochemistry parameters of total cholesterol (T-CH) and total triglycerides (TG) (*P* = 0.057). As to high-density lipoprotein cholesterol (HDL-CH) and low-density lipoprotein cholesterol (LDL-CH), G group resulted in a greater HDL-CH level than B group (*P* = 0.002), whereas B group resulted in a greater LDL-CH level than G group (*P* = 0.017). Furthermore, the G-B treatment significantly increased the birds’ glucose (GLU) level (*P* = 0.003) in comparison with the G and the G × B treatments (Fig. 7). Additionally, there was no effect of the G, G × B, B, or F treatments on the GLU level (*P* = 0.820).

**Figure 8:**
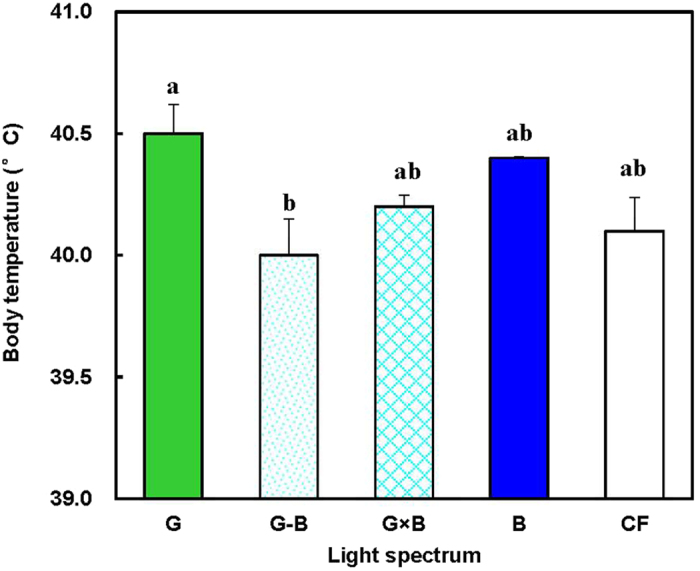
Body temperature (°C) of 81-day-old broilers reared under different lighting system. Each group of treated birds was reared under either cell (replicate) equipped with two green LED lights (G group), cell (replicate) equipped with a green LED light and a blue LED light (G-B group), cell (replicate) equipped with two green-blue-dual-wavelength LED lights (G × B group), cell (replicate) equipped with two blue LED lights (B group), or cell (replicate) equipped with two normal artificial lights (F group as control group; fluorescent lamps) from 1 day old until termination of experiment at 81 day of age. At the end of the trial (81 day of age), body temperature was measured using an infrared thermometer. Data are expressed as mean value ± SEM. Bars marked with different letters are significantly different from each other (*P* < 0.05).

The birds in the G treatment had a significantly higher body temperature (°C) than the birds in the G-B treatment (*P* = 0.035) (Fig. 8). However, no differences were found among the G, G × B, B, and F groups (*P* = 0.153).

## Discussion

Previous studies have suggested that stress and environmental factors were of great importance in poultry production^16^. Poultry rely heavily on visual cues when judging what is safe to eat and drink^17^ and the appropriate feeding behavior is facilitated by an innate predisposition to peck at small particles and flat shiny surfaces^18^,^19^,^20^. Moreover, vision is most likely the dominant sense in domestic poultry because the majority of their behavior is mediated by vision. Therefore, light plays a vital role in affecting the growth of chicken. In the present study, as the same with our previous study, we found the green LED light and blue LED light stimulates the growth of birds compared with normal white light^15^. Moreover, we found that the effect of the green/blue mixed LED light (G-B and G × B groups) have the same stimulatory effect on broiler body weight with single green light and single light (G and B groups), compared with normal artificial light (F group). Additionally, no significant differences were found the body weight of birds between the green/blue mixed LED light (G-B and G × B groups) and single green light or single blue light. Moreover, the percentage of carcass was significantly greater in the green/blue mixed light (G × B group) when compared with single blue light (B group).

A previous study indicated that single green light promoted early age growth by enhancing the proliferation of skeletal muscle satellite cells^21^. Moreover, the expression of a growth hormone receptor gene was also higher in the green light groups^21^. Another study found that green light applied during embryogenesis enhanced the proliferation of myofibers and influenced myofiber growth by increasing the number of uniform myofibers^22^. Although single blue light had less impact on early age muscle growth, blue light was reported to increase plasma androgens in older birds^23^. Moreover, single blue light may play a role in alleviating the stress response in broilers^24^. Accordingly, when birds were exposed to green light at an early age and then those birds were shifted to blue light, the birds’ growth was further promoted^25^. In present study, we found that the mixture of green and blue single lights combine the advantages of the two wavelengths, and indicated a stimulatory effect when both green light and blue light were applied to the chicken simultaneously. In addition, blue light enhanced protein synthesis and reduced protein breakdown^26^,^27^,^28^ through an increase in plasma androgens^23^. However, we suggested that the blue light might reduce adipose synthesis or metabolism because of the significant decrease in the abdominal adipose weight in the single blue light in present study. In present study, we used lux as intensity unity in the present study. First, previous research in our laboratory and other studies^29^,^30^ have shown that light intensity has no influence on chicken growth, which confirmed that the differences in growth performance of light-treated birds were caused by the light spectrum rather than light intensity. Second, long-wavelength light contain more energy and are able to penetrate through the skull and brain tissue to easily stimulate the hypothalamus than short-wavelength light^31^. Therefore, it was suggested that short-wavelength (blue/green light) require higher intensities to stimulate hypothalamic photoreceptors. The present light intensity is very low (15 lux). According to the domestic fowl’s spectral sensitivity curve^32^, the adjusted measurement is still very low. Third, it is not practical to use a spectrometer to measure light intensity and adjust the measurement. A spectrometer (usually $109,040 ~ $20,060) is substantially more expensive than a lux radiometer (usually $100 ~ $160).

Similarly, the green/blue mixed light (G-B and G × B groups) had a significant improved influence on the feed conversion of birds compared with normal artificial light (F group). Moreover, at the market age of bird (81 days), a significant improvement in feed conversion were found in green/blue mixed light (G × B group) compared with single LED light (G group and B group). Previous study indicated that single blue light improved small intestine function^33^. Broilers were reported to be more active (more walking) in white light than in green or blue light, which can affect the behavior of broilers^34^. In addition, blue light can be used to reduce the activity of the birds^35^. In the present study, the lower feed conversion and the resting condition of the broilers reared under the blue lights in combination (G × B group) or alone and (B group) may be explained by the calming influence of blue light on broilers.

Light allows the bird to establish rhythmicity and to synchronize many essential functions, including body temperature and various metabolic steps that facilitate feeding and digestion^36^. In our study, we found that single green light (G group) resulted in a greater HDL-CH level than single blue light (B group). On the contrary, single blue light (B group) resulted in a greater LDL-CH level than single green light (G group). However, there were no significant differences found between the green/blue mixed light (G-B and G × B groups) and the single light (G and B groups) for the main blood biochemistry parameters of total cholesterol (T-CH), total triglycerides (TG), high-density lipoprotein cholesterol (HDL-CH), and low-density lipoprotein cholesterol (LDL-CH), which indicates that the green/blue mixed light increase the growth of birds without causing the change of the main blood biochemistry parameters. As to glucose (GLU) level, the green/blue mixed light (G × B group) significantly increased the birds’ GLU level in comparison with the single light (G group).

In our study we also found that the birds in the single light (G group) had a significantly higher body temperature (°C) than the birds in the green/blue mixed light (G-B group). It is well documented that many aspects of the health, productivity and well-being of the animals may be at some risk from stress in those birds without a normal body temperature^37^,^38^. The chicken is an endothermic animal, and the regulation of body temperature in endothermic animals is achieved in two ways, i.e., the autonomic and the behavioral mechanisms^39^. The autonomic response is generated by an endogenous component controlled by a circadian clock, and the behavioral response is generated by an exogenous component mainly driven by variation in motor activity^40^,^41^,^42^. These two functions are related metabolically and temporally^40^. The significant difference in body temperature between the single light and the green/blue mixed light in our experiment suggested that the body temperature regulation was affected by the light spectrum. The light spectrum might alter the physiology of the birds by releasing the thyroid hormone^43^ or might alter their behavior by changing the level of motor activity^44^ to maintain body temperature.

In conclusion, a new method to manipulate chick growth and metabolism has been introduced by mixing the growth-advantage LED (monochromatic green LED light and blue LED light). First, the green LED chip and blue LED chip have been combined to fabricate the mixed LED arrays; second, the chicks exposed to a mixture of green and blue single lights (green/blue mixed light) attained a stimulatory effects on body weight than birds exposed to normal artificial light without resulting in the blood biochemistry parameters fluctuation. Moreover, the percentage of carcass was significantly greater in the green/blue mixed light (G × B group) when compared with single blue light (B group). At the market age of bird (81 days), a significant improvement in feed conversion were found in green/blue mixed light (G × B group) compared with single LED light (G group and B group). Present results suggested that the growth and metabolism of broiler can be affected by the green/blue mixed light, which might establish an effective strategy for maximizing the growth of broilers by using mixed LED technology. Therefore, further study should be designed to optimize the ratio of green component to blue component of the mixed light for the optimal broiler production.

## Methods

All experimental protocols were approved by the committee of the Care and Use of Animals of the Zhejiang University. The methods were carried out in strict accordance with the guidelines of the Association for the Study of Animal Behaviour Use of the Zhejiang University.

### Animals and experimental design

The present study was carried out in Yuhang Qingqin Poultry Farm, which is located within Hangzhou zone between latitude 30.26°and longitude 120.19° as our previous study^14^. Because of its tender, delicious and nutritious meat, the Chinese-native “*Meihuang*” broiler is very popular in China as described in our previous study^10^. Therefore, in this study, 300 Chinese-native female broiler chickens (“*Meihuang*”; age, 0 day; average body weight, 30.5 ± 0.3 g) were used that were purchased from a commercial hatchery (Guangda Breeding Co. Ltd., China). On day 0, all broilers were randomly housed in 5 light treatment (60 birds per light treatment). Each treatment consisted of four replicate pens containing 15 broiler chicks each in a light tight room outfitted with one of the light sources. Four replicate galvanized wire pens (1 m × 1 m × 1 m, length × width × height) were placed 0.25 m above the ground. The bottom of the cage were covered with mesh. A solid board covered with wood-shavings was placed 0.25 m below the cage to collect manure. The wood-shavings were replaced at weekly intervals (3 days). Each of the five rooms utilized was set up in an identical pattern, with the only difference being the light bulbs in the fixtures. Each pen was surrounded and covered with fluorescent fabrics to avoid light pollution from other sources.

**Figure 9:**
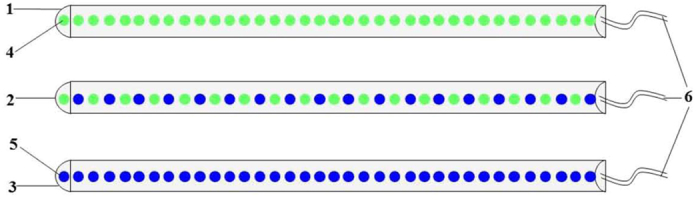
The detailed information of the LED lighting systems used in this study. 1. green LED lighting which was fabricated with 36 green LED chips (4); 2. dual-wavelength LED lighting which was fabricated with 18 green LED chips and 18 blue LED chips (5); 3. blue LED lighting which was fabricated with 36 blue LED chips. 4. green LED chips (514 nm); 5. blue LED chips (415 nm); 6. power line.

Each group of treated broilers was exposed to the following 5 light treatments: (1) the replicate cells were equipped with two green LED lights (G group), (2) the replicate cells were equipped with a green LED light and a blue LED light (G-B group), (3) the replicate cells were equipped with two green-blue-dual-wavelength LED lights (G × B group), (4) the replicate cells were equipped with two blue LED lights (B group), and (5) the replicate cells were equipped with two normal artificial lights (F group as control group; fluorescent lamps). The detailed information for the LED light system is presented in Fig. 9. The LED light sources were purchased from the Langtuo Biological Technology Co. Ltd. (Hangzhou, China). A total of 36 LED chips were installed linearly on a board to fabricate the LED lighting system. Each group of lamps were placed 75 cm above the broilers using plastic ties that were attached to the ceiling. Basically, pulse width modulation (PWM) uses a driving current that is determined from the peak current, period of repetition, and pulse duty. The pulse duty is the ratio of ON time to the period that controls the average light intensity. Therefore, in this study, we used the PWM method to control the light intensity precisely and a radiometer (AR823, Digital Lux Meter Co. Ltd., China) to maintain the intensity with 15 lux as our previous study^9^. The light schedule was 23 h light: 1 h dark on the first day as the birds adapted to the environment and then was reduced by one hour every day until it reached 16 h light: 8 h dark, which was maintained for the remainder of the study.

Each of the light treated birds had similar initial body weights: 30.1 ± 0.3 (G group), 30.4 ± 0.2 g (G-B group), 30.5 ± 0.2 (G × B group), and 30.2 ± 0.5 (B group), and 30.4 ± 0.3 (F group). When the brooding period ended (day 30), all birds were weighted individually and the average body weight was calculated immediately for each light treatment. Then, five birds (for example, the lame or malnutrition birds) were eliminated from each replicate to maintain uniformity without creating a deviation from the original average body weight. The dry bulb temperature and relative humidity were measured once each day using data loggers (TH602F, Anymetre Co. Ltd., China) to ensure that the temperature and relative humidity conditions were similar in all rooms. The average temperature and relative humidity was 25.3 °C and 67.5%. These conditions were maintained by an electric heater thermostat and a ventilator. The broilers had *ad libitum* access to feed and water. All broilers were fed with the same starter diet (13.4 MJ ME/kg; 220 g/kg crude protein (CP)) when they were between 1 and 21 days old, followed by a grower diet (13.6 MJ ME/kg; 200 g/kg CP) until the end of the experiment (81 days old).

### Growth performance collection

It is very technically challenging for us to measure early body weights. Young chickens are highly vulnerable to stress and injury. Obtaining the body weight of young chicks would require that we catch the chicks from their pens and weigh them individually. Such handling can cause undue distress to these animals and could result in injuries or even death. In addition, it is our experience that when we weigh young chicks, the data obtained using an electronic balance fluctuate severely, a fact that prevents us from obtaining precise body weight values. In contrast, such operations are feasible in older chickens. Therefore, in this study the early body weights (before day 30) were not obtained. The body weight was recorded on day 30, 45, 60, 72 and 81. The daily feed consumption was measured, and the feed efficiency was calculated.

### Metabolic indicators collection

On day 81, the body temperatures of birds were measured using an infrared thermometer (No. 303B, Victor Co. Ltd., China). At the end of the trial (81 d), after fasting for 12 h, three broilers from each replicate were randomly selected from each treatment, maintaining the same number of birds across all replicates and treatments, for a total of 60 broilers (3 birds/replicate × 4 replicates × 5 treatments). The selected broilers were killed by cervical dislocation to collect blood samples and eviscerated to measure the weights of the heart, the liver, the gizzard, the spleen and the abdominal adipose tissue. From each broiler, a 5 ml sample of blood was obtained. The blood samples were centrifuged at 4 °C for 30 min at 3,000  × g to remove any clots. The blood serum was aspirated and stored in sealed polypropylene microcentrifuge tubes at −70 °C for subsequent determination. The metabolic indicators total cholesterol (TC), total triglycerides (TG), high-density lipoprotein cholesterol (HDL-CH), low-density lipoprotein cholesterol (LDL-CH) and glucose (GLU) were determined using an Automatic Biochemistry Analyzer (AU5400, Olympus Co. Ltd., Japan). The conditions on the slaughter line were designed to reduce the birds’ stress.

### Statistics analysis

Data were subjected to statistical analyses using the SPSS Statistical (V. 20) software package. An ANOVA was performed to analyze the effects of the different lights on the broilers. The homogeneity of variance was checked for each set of data, and no transformations were required. When appropriate, post hoc comparisons were made using the least significant difference test. The data are presented as the average ± SEM. In every case, differences between the groups were considered statistically significant if the value of *P* < 0.05.

## Additional Information

**How to cite this article**: Yang, Y. *et al.* A new method to manipulate broiler chicken growth and metabolism: Response to mixed LED light system. *Sci. Rep.* **6**, 25972; doi: 10.1038/srep25972 (2016).
